# Distinct NGS mutational landscape and prognostic implications in early-onset colorectal cancer: A dual-cohort analysis of TCGA PanCancer Atlas and MSK-IMPACT 50K

**DOI:** 10.1371/journal.pone.0359094

**Published:** 2026-10-01

**Authors:** Elif Sertesen Çamöz, Osman Bilge Kaya, Hakan Erçelebi, Cengiz Karaçin, Yunus Kasım Terzi, Zerrin Yılmaz Çelik

**Affiliations:** 1 Department of Medical Oncology, Dr. Abdurrahman Yurtaslan Ankara Oncology Training and Research Hospital, University of Health Sciences, Ankara, Turkey; 2 Department of Medical Genetics, Başkent University, Ankara, Turkey; 3 Department of Pediatric Neurology, Başkent University, Ankara, Turkey; Osaka International Cancer Institute: Osaka Kokusai Gan Center, JAPAN

## Abstract

**Background:**

Early-onset colorectal cancer (EOCRC), defined as CRC diagnosed before age 50, is rising globally. The genomic landscape of EOCRC has been characterized in several prior studies, but the reproducibility of findings across independent cohorts, the impact of adjustment for tumor stage and sample type, and the specificity of prognostic biomarkers to the EOCRC subgroup remain incompletely defined. Furthermore, BRAF mutation frequency is strongly modified by tumor location, which is often not considered in prior analyses.

**Methods:**

We performed a dual-cohort analysis using two independent, publicly available genomic databases: The Cancer Genome Atlas (TCGA) PanCancer Atlas (discovery cohort) and the MSK-IMPACT 50K Clinical Sequencing Cohort (validation cohort). Patients with any somatic mutation in the four canonical MMR genes (MLH1, MSH2, MSH6, PMS2) were excluded to reduce the influence of MMR-deficient tumors on comparisons. After exclusion, the discovery cohort comprised 536 CRC patients (EOCRC n = 67, LOCRC n = 469) and the validation cohort comprised 4,254 CRC patients (EOCRC n = 1,470, LOCRC n = 2,784). Somatic mutation frequencies for 11 genes and MSI status were compared between EOCRC and LOCRC, with BRAF analyses further stratified by BRAF V600E specifically and by tumor location. Kaplan-Meier overall survival (OS) analyses were performed with number-at-risk tables. Multivariate Cox regression was adjusted for tumor stage (TCGA) or sample type (Primary vs. Metastasis; MSK-IMPACT). An EOCRC-specific multivariate model was also constructed to test whether prognostic effects were specific to the young-onset subgroup.

**Results:**

BRAF total mutations were significantly less frequent in EOCRC in the validation cohort (6.5% vs 10.1%, p < 0.001); BRAF V600E specifically showed a similar pattern (3.3% vs 7.5%, p < 0.001). However, stratification by tumor location attenuated this difference substantially, indicating that BRAF depletion in EOCRC is at least partially explained by differential tumor location distribution. Kaplan-Meier median OS in the TCGA cohort was not reached for EOCRC and 81.4 months for LOCRC (log-rank p = 0.212); in the MSK-IMPACT cohort, EOCRC 49.4 vs LOCRC 44.4 months (p = 0.009). Multivariate Cox regression in the MSK-IMPACT cohort adjusted for sample type identified KRAS (HR = 1.23, p < 0.001), NRAS (HR = 1.38, p = 0.009), and BRAF (HR = 1.29, p = 0.005) as independent adverse prognostic factors; MSI-H was protective (HR = 0.55, p = 0.008); metastatic sample was a strong independent predictor (HR = 1.70, p < 0.001). Age group was not independently prognostic. In the EOCRC-specific analysis, KRAS, NRAS, and BRAF mutations were not independently prognostic within EOCRC.

**Conclusions:**

After exclusion of MMR-deficient tumors, tumor location adjustment, and sample type adjustment, BRAF mutation depletion in EOCRC is confirmed but is at least partially explained by tumor location distribution differences. KRAS/NRAS/BRAF and MSI-H are validated as CRC prognostic biomarkers, but these effects are driven by the overall cohort and are not specific to the EOCRC subgroup. These findings support comprehensive NGS profiling in EOCRC while cautioning against interpreting general CRC prognostic biomarkers as EOCRC-specific.

## 1. Introduction

Colorectal cancer (CRC) is the third most commonly diagnosed malignancy and the second leading cause of cancer-related mortality worldwide [1]. A particularly concerning epidemiological trend is the rising incidence of early-onset colorectal cancer (EOCRC), defined as CRC diagnosed before age 50, which has increased by approximately 2% annually in recent decades despite stable or declining rates in older populations [2,3]. EOCRC now accounts for approximately 10–12% of all CRC diagnoses in high-income countries [4].

EOCRC is increasingly recognized as clinically and biologically distinct from late-onset CRC (LOCRC, diagnosed at age ≥ 50 years). EOCRC patients often present at more advanced disease stages, more commonly harbor rectal or left-sided tumors, and are frequently diagnosed outside conventional screening programs [5,6]. Understanding the genomic landscape of EOCRC is clinically actionable: it may identify targetable alterations, inform immunotherapy eligibility, and guide prognosis in a population for whom standard prognostic models were not developed.

Several important prior studies have characterized the mutational landscape of EOCRC. Systematic review evidence and multicohort analyses have consistently identified lower rates of BRAF mutation in EOCRC compared to LOCRC [7–10]. However, three important limitations of prior work remain incompletely addressed. First, most single-database analyses do not include an independent validation cohort with substantially larger EOCRC sample size for replication. Second, BRAF mutation frequency in CRC is strongly modified by tumor location, being highly enriched in right-sided (proximal) colon tumors, and EOCRC patients disproportionately have distal or rectal tumors; therefore, BRAF depletion in EOCRC may partially reflect tumor location distribution rather than an age-onset-specific biological difference. Third, whether well-established CRC prognostic biomarkers (e.g., KRAS, NRAS, BRAF, MSI-H) retain independent prognostic value specifically within the EOCRC subgroup has not been rigorously tested.

To address these gaps, we conducted a pre-specified dual-cohort analysis. We selected the TCGA PanCancer Atlas as the discovery cohort because it is the most widely used reference genomic dataset in cancer research, providing harmonized somatic mutation, tumor stage, and outcome data in a population-based multi-institutional research setting. We selected the MSK-IMPACT 50K Clinical Sequencing Cohort as the validation cohort because it represents the largest publicly available real-world clinical NGS dataset (>50,000 patients), includes a substantially larger EOCRC subgroup (n = 1,629) than any prior single-database analysis, and was generated in a clinical rather than research setting, providing an independent, complementary perspective. Other publicly available CRC databases were considered but excluded due to insufficient survival data, absence of gene-level NGS mutation profiling, or substantially smaller sample sizes. Our analysis is specifically designed to (i) exclude MMR-deficient tumors via somatic MMR gene mutation status, (ii) distinguish BRAF V600E from other BRAF mutations, (iii) stratify BRAF analyses by tumor location, (iv) adjust survival models for stage (TCGA) and sample type (MSK-IMPACT), and (v) test whether observed prognostic effects are specific to EOCRC.

## 2. Materials and methods

### 2.1. Data sources and patient selection

Two independent publicly available cohorts were analyzed via the cBioPortal for Cancer Genomics REST API (https://www.cbioportal.org) [11,12]. The discovery cohort was derived from The Cancer Genome Atlas (TCGA) PanCancer Atlas colorectal adenocarcinoma dataset (COAD and READ). The validation cohort was derived from the MSK-IMPACT 50K Clinical Sequencing Cohort. Both datasets are fully de-identified and publicly available under open-access data use agreements; no institutional ethics approval was required for this secondary analysis. Data access date: April 2026.

Inclusion criteria were: colorectal adenocarcinoma (COAD, READ, or COADREAD by Oncotree code), available age at diagnosis, and available somatic mutation profile. Patients were classified as EOCRC (age < 50 years) or LOCRC (age ≥ 50 years) at diagnosis, with LOCRC serving as the reference category throughout all comparisons. To reduce the influence of MMR-deficient tumors on genomic and prognostic comparisons, we excluded all patients with any somatic mutation in the four canonical MMR genes: MLH1, MSH2, MSH6, or PMS2. This exclusion captures Lynch-associated cases through the two-hit mechanism (in which a germline MMR mutation is accompanied by a somatic second-hit mutation in the tumor, which is detected in tumor-only somatic sequencing) as well as sporadic MMR-mutant cases. This is a conservative approach that captures both hereditary and somatic MMR-driven tumors; however, we note that MSI-H tumors caused by sporadic MLH1 promoter hypermethylation (without MMR gene mutation) remain in the analysis cohort, as methylation data are not available in cBioPortal for these studies.

### 2.2. Genomic data extraction

Somatic mutation data were retrieved from the mutation profile of each study. Both datasets use tumor-normal paired sequencing to identify somatic variants: in TCGA, germline variants are filtered by paired-normal whole-exome sequencing; in MSK-IMPACT, tumor-normal paired panel sequencing is used. Eleven genes were selected a priori based on established clinical relevance in CRC: KRAS, NRAS, BRAF, TP53, PIK3CA, APC, SMAD4, CTNNB1, ERBB2, POLE, and POLD1. In addition, MMR genes (MLH1, MSH2, MSH6, PMS2) were extracted for MMR-deficient tumor exclusion. A binary mutation status (mutated/wild-type) was assigned per gene per patient. For BRAF, we additionally extracted protein change annotations to identify the specific V600E hotspot mutation (and its alternative reference position V640E when annotated as such); BRAF V600E was analyzed as a separate variable in addition to BRAF (any). MSI status in the TCGA cohort was derived from molecular subtype annotations (MSI subtypes classified as MSI-H). MSI status in the MSK-IMPACT cohort was derived from the MSI_TYPE clinical attribute (Instable = MSI-H). Tumor location was categorized based on ICD-O-3 site code (TCGA) or PRIMARY_SITE annotation (MSK-IMPACT) as proximal colon (cecum through transverse colon), distal colon (descending and sigmoid), rectum (including rectosigmoid), or unspecified.

Tumor stage was extracted from AJCC_PATHOLOGIC_TUMOR_STAGE in TCGA and simplified into groups I, II, III, IV. Stage III-IV was analyzed as “Stage advanced” in multivariate models. AJCC pathologic stage was not available in the MSK-IMPACT cohort. However, sample type (Primary vs. Metastasis vs. Local Recurrence) is universally documented in MSK-IMPACT and was used as a stage-related proxy in multivariate models: this variable captures whether the sequenced specimen was obtained from a metastatic or recurrent site, which correlates strongly with disease stage.

### 2.3. Statistical analysis

Mutation frequency comparisons between EOCRC and LOCRC were performed using chi-square tests or Fisher’s exact test where appropriate (expected cell count <5). All reported p-values for mutation frequency comparisons are nominal and uncorrected for multiple comparisons; given the pre-specified clinically relevant gene panel, we report nominal p-values but note that 13 simultaneous comparisons were performed per cohort. BRAF stratification analyses were performed within each tumor location subgroup.

Overall survival (OS) was measured differently in each cohort reflecting available data: in the TCGA cohort, OS was calculated from the date of initial pathologic diagnosis; in the MSK-IMPACT cohort, OS was calculated from the date of first sample collection. This difference is an important methodological limitation that introduces potential lead-time and immortal-time bias in the MSK-IMPACT cohort, because sequencing in a tertiary clinical setting is typically performed at the time of recurrence, progression, or metastatic biopsy. We explicitly reframe MSK-IMPACT survival analysis as “survival following clinical molecular sequencing” rather than natural disease-onset survival. Direct cross-cohort survival comparisons are not appropriate and were not performed. Kaplan-Meier median OS is reported using the standard Kaplan-Meier estimator (time at which the survival curve first drops below 0.5). Number-at-risk tables are provided for all Kaplan-Meier figures.

Univariate Cox proportional hazard regression was performed for each variable. Variables with p < 0.10 in univariate analysis (or of a priori clinical interest) were candidates for multivariate models. Multivariate Cox models were constructed separately for each cohort. In the TCGA multivariate model, tumor stage (Stage III-IV vs. I-II) was included as a covariate. Patients with missing stage data (n = 10; 1.9% of the TCGA analysis cohort) were excluded from the TCGA multivariate Cox model (complete-case analysis). In the MSK-IMPACT multivariate model, sample type (Metastatic vs. Primary) was included as a stage-related covariate. An EOCRC-restricted multivariate model was also constructed in the MSK-IMPACT cohort to test whether biomarker effects observed in the full cohort remained independent within EOCRC alone. The proportional hazards assumption was evaluated using Schoenfeld residual analysis; no significant violations were identified. Missing OS data were excluded from survival analyses.

All statistical analyses were performed using Python (version 3.12) with NumPy, SciPy, and custom Cox partial likelihood optimization. Figures were generated using Matplotlib. The authors used Claude (Anthropic, claude-sonnet-4-6) to assist with data extraction, statistical computations, manuscript drafting, and figure generation. The authors have reviewed and edited all outputs and take full responsibility for the content of this publication.

## 3. Results

### 3.1. Patient characteristics

After exclusion of MMR-mutation-positive patients (56 in TCGA, 402 in MSK-IMPACT), the final analysis cohorts comprised: TCGA discovery cohort n = 536 (EOCRC n = 67; LOCRC n = 469) and MSK-IMPACT validation cohort n = 4,254 (EOCRC n = 1,470; LOCRC n = 2,784). Clinical and demographic characteristics are summarized in Table 1.

**Table 1:** Clinical characteristics after MMR-mutated tumor exclusion.

Variable	TCGA EOCRC (n = 67)	TCGA LOCRC (n = 469)	p	MSK EOCRC (n = 1,470)	MSK LOCRC (n = 2,784)	p
Age, median (IQR), yrs	45 (41-48)	69 (62-77)	—	43 (37-46)	61 (55-68)	—
Sex: Male, n (%)	27 (40.3%)	254 (54.2%)	0.046*	757 (51.5%)	1,544 (55.5%)	0.015*
Tumor location: proximal	15 (22.4%)	159 (33.9%)	—	133 (9.0%)	404 (14.5%)	—
Tumor location: distal	17 (25.4%)	107 (22.8%)	—	334 (22.7%)	451 (16.2%)	—
Tumor location: rectum	22 (32.8%)	122 (26.0%)	—	400 (27.2%)	636 (22.8%)	—
Stage III-IV (TCGA)	42 (62.7%)	200 (42.6%)	0.003*	N/A	N/A	—
Metastatic sample (MSK)	N/A	N/A	—	435 (29.6%)	811 (29.1%)	0.780
Deaths, n (%)	9 (13.4%)	98 (20.9%)	—	458 (31.2%)	947 (34.0%)	—
Median OS (KM), months†	NR	81.4	0.212	49.4	44.4	0.009*

EOCRC: early-onset CRC (<50y); LOCRC: late-onset CRC (≥50y, reference category); IQR: interquartile range; NR: not reached. *p < 0.05. †OS measured from date of diagnosis (TCGA) or date of sample collection (MSK-IMPACT); direct cross-cohort comparison is not appropriate. Median OS estimated by Kaplan-Meier method.

Median age at diagnosis was 45 years (IQR 41–48) for EOCRC and 69 years (IQR 62–77) for LOCRC in the TCGA cohort, and 43 years (IQR 37–46) for EOCRC and 61 years (IQR 55–68) for LOCRC in the MSK-IMPACT cohort. Male sex was less frequent among EOCRC patients in both cohorts (TCGA: 40.3% vs 54.2%, p = 0.046; MSK-IMPACT: 51.5% vs 55.5%, p = 0.015). Tumor location differed by age group: EOCRC patients had proportionally fewer proximal tumors and more rectal tumors compared to LOCRC in both cohorts. In the TCGA cohort where AJCC stage was available for 98.1% of patients, EOCRC patients presented with substantially more advanced disease: 62.7% of EOCRC vs 42.6% of LOCRC had Stage III-IV disease (p = 0.003). In the MSK-IMPACT cohort, the proportion of metastatic samples was similar between EOCRC and LOCRC (29.6% vs 29.1%, p = 0.78).

### 3.2. Somatic mutation frequencies

Somatic mutation frequencies are presented in Table 2 and Fig 1. In the MSK-IMPACT validation cohort, several statistically significant differences were observed between EOCRC and LOCRC. BRAF mutations (any) were less frequent in EOCRC (6.5% vs 10.1%, p < 0.001), and BRAF V600E specifically was markedly less frequent (3.3% vs 7.5%, p < 0.001). TP53 mutations were more frequent in EOCRC (82.5% vs 75.2%, p < 0.001). KRAS mutations were less frequent in EOCRC (41.8% vs 45.5%, p = 0.022). PIK3CA mutations were less frequent (16.5% vs 19.4%, p = 0.024). MSI-H rates were lower in EOCRC (2.3% vs 4.4%, p = 0.001), a finding that emerged only after MMR-deficient tumor exclusion, because pre-exclusion MSI-H rates are dominated by cases with a detectable MMR gene mutation, many of which are Lynch-associated. CTNNB1 showed a marginal trend toward differential frequency in both cohorts but did not achieve statistical significance (TCGA: 9.0% vs 3.4%, p = 0.070; MSK: 4.5% vs 5.8%, p = 0.079). Findings in the TCGA discovery cohort were directionally consistent for BRAF and CTNNB1 but did not reach statistical significance, an expected limitation of the small TCGA EOCRC subgroup (n = 67).

**Table 2:** Somatic mutation frequencies in EOCRC vs LOCRC (MMR-mutated tumors excluded).

Gene/Marker	TCGA EOCRC (n = 67)	TCGA LOCRC (n = 469)	p	MSK EOCRC (n = 1,470)	MSK LOCRC (n = 2,784)	p
**KRAS**	24 (35.8%)	173 (36.9%)	0.973	615 (41.8%)	1,268 (45.5%)	0.022*
**NRAS**	2 (3.0%)	27 (5.8%)	0.562	53 (3.6%)	117 (4.2%)	0.388
**BRAF (any)**	4 (6.0%)	37 (7.9%)	0.806	96 (6.5%)	282 (10.1%)	<0.001*
**BRAF V600E**	2 (3.0%)	30 (6.4%)	0.408	49 (3.3%)	210 (7.5%)	<0.001*
**TP53**	39 (58.2%)	245 (52.2%)	0.432	1,213 (82.5%)	2,094 (75.2%)	<0.001*
**PIK3CA**	13 (19.4%)	111 (23.7%)	0.536	243 (16.5%)	540 (19.4%)	0.024*
**APC**	39 (58.2%)	311 (66.3%)	0.244	1,145 (77.9%)	2,129 (76.5%)	0.314
**SMAD4**	7 (10.4%)	51 (10.9%)	1.000	211 (14.4%)	405 (14.5%)	0.901
**CTNNB1**	6 (9.0%)	16 (3.4%)	0.070‡	66 (4.5%)	162 (5.8%)	0.079‡
**ERBB2**	0 (0.0%)	14 (3.0%)	0.235	46 (3.1%)	99 (3.6%)	0.522
**POLE**	3 (4.5%)	12 (2.6%)	0.417	33 (2.2%)	80 (2.9%)	0.266
**POLD1**	1 (1.5%)	14 (3.0%)	0.707	22 (1.5%)	52 (1.9%)	0.449
**MSI-H**	4 (6.0%)	30 (6.4%)	1.000	34 (2.3%)	123 (4.4%)	0.001*

All p-values nominal, uncorrected for multiple comparisons. ‡p < 0.10 (trend). *p < 0.05. Chi-square or Fisher’s exact test. LOCRC (≥50y) serves as reference category.

**Fig 1:**
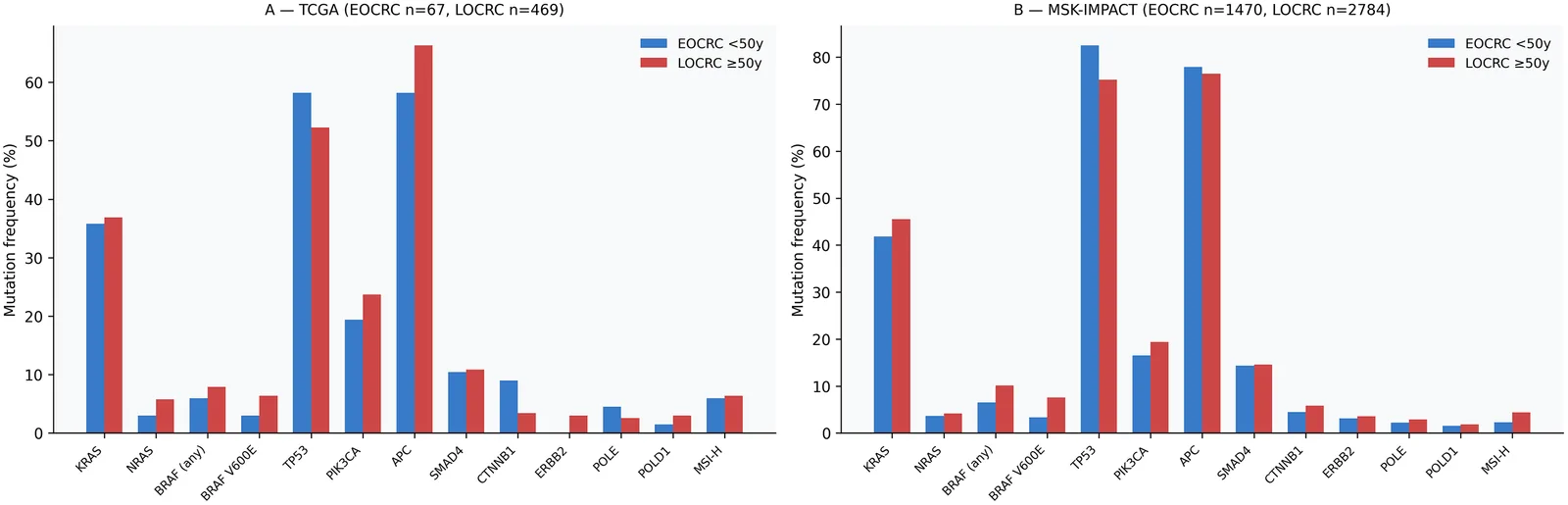
Somatic mutation frequencies (%) for 11 genes plus BRAF V600E specifically and MSI-H in EOCRC vs LOCRC. Left: TCGA discovery cohort. Right: MSK-IMPACT validation cohort. MMR-mutated tumors excluded.

### 3.3. BRAF mutation frequency by tumor location

Because BRAF mutation frequency in CRC is well-known to vary strongly by anatomic location, and because EOCRC patients have a different tumor location distribution than LOCRC, we stratified BRAF analyses by tumor location (Fig 2). Within each tumor location subgroup (proximal, distal, rectum), the differences in BRAF frequency between EOCRC and LOCRC were substantially attenuated: in the MSK-IMPACT validation cohort, proximal EOCRC vs LOCRC BRAF was 15.0% vs 20.3% (p = 0.225); distal EOCRC vs LOCRC was 6.6% vs 6.0% (p = 0.85); rectal EOCRC vs LOCRC was 4.0% vs 5.2% (p = 0.47). This finding is consistent with the interpretation that the age-related BRAF differences observed in unstratified comparisons are at least partially explained by the differential distribution of tumor location between age groups. This has important implications for the biological interpretation of BRAF-EOCRC associations reported in prior single-database analyses that did not adjust for tumor location.

**Fig 2:**
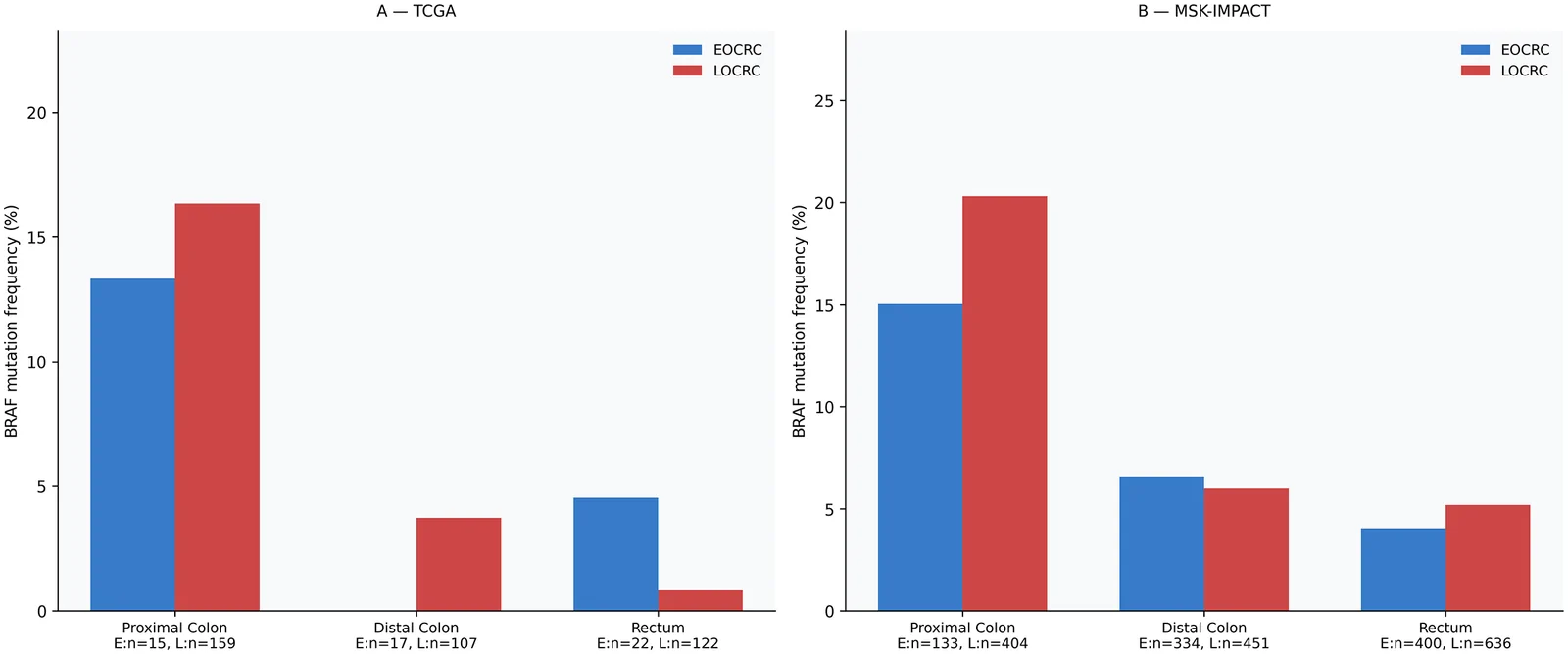
BRAF mutation frequency by tumor location (proximal colon, distal colon, rectum) in EOCRC vs LOCRC. Left: TCGA. Right: MSK-IMPACT. Within-location BRAF differences are substantially smaller than unstratified comparisons, indicating that BRAF-EOCRC associations are at least partially explained by differential tumor location distribution.

### 3.4. Overall survival

Kaplan-Meier survival curves with number-at-risk tables are shown in Fig 3. In the TCGA discovery cohort (measuring OS from date of diagnosis), median OS was not reached (NR) for EOCRC (9 events among 65 patients with survival data) and was 81.4 months for LOCRC (94 events among 448 patients with survival data; log-rank p = 0.212). In the MSK-IMPACT validation cohort (measuring OS from date of sample collection), median OS was 49.4 months for EOCRC and 44.4 months for LOCRC (log-rank p = 0.009). The apparently favorable OS of EOCRC in the MSK-IMPACT cohort should be interpreted with the strongest caution given the risk of lead-time and immortal-time bias inherent in the sample-collection OS definition, as discussed below. The two cohorts measure OS from different origins and are not directly comparable.

**Fig 3:**
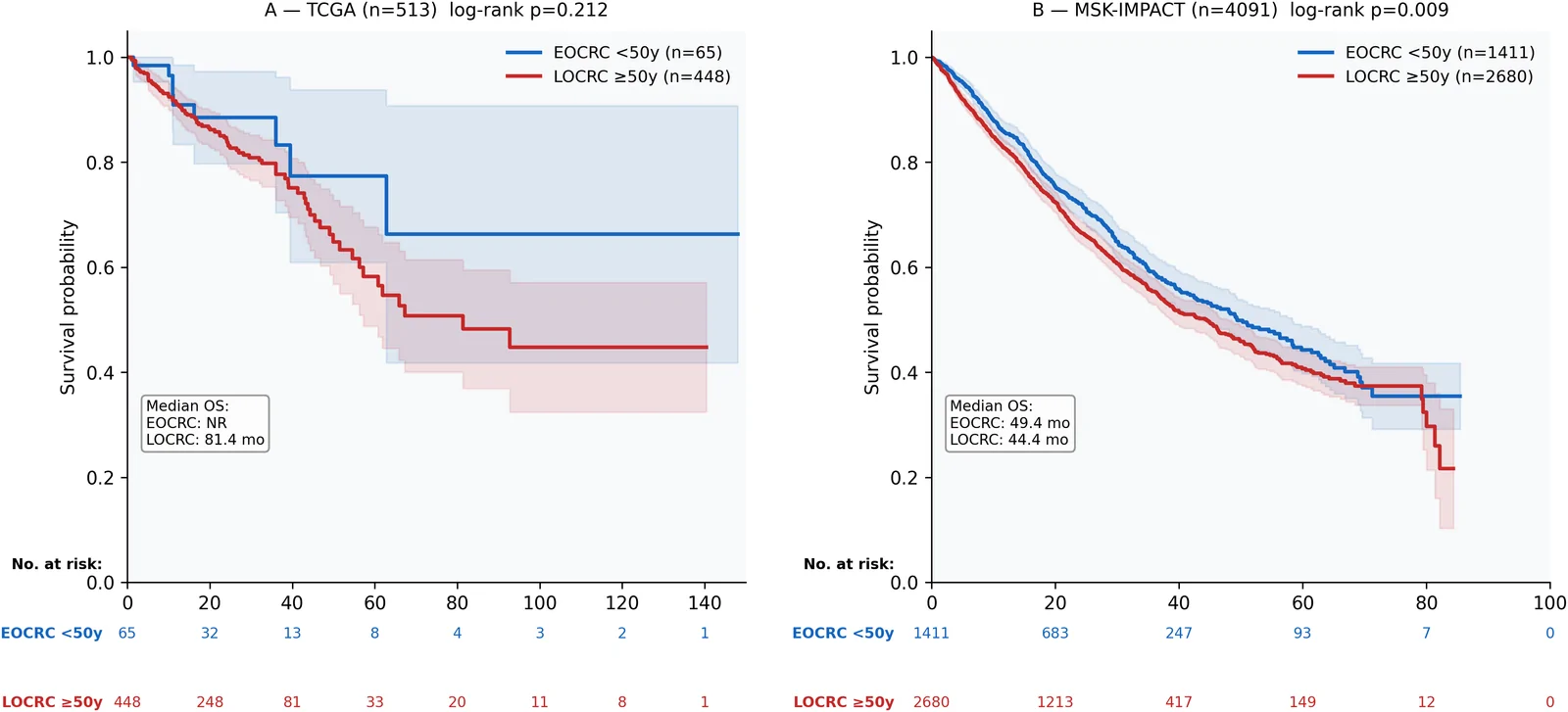
Kaplan-Meier overall survival curves with number-at-risk tables in EOCRC vs LOCRC after MMR-mutated tumor exclusion. Left: TCGA discovery cohort (OS from date of diagnosis; median OS EOCRC not reached, LOCRC 81.4 months). Right: MSK-IMPACT validation cohort (OS from date of sample collection; median OS EOCRC 49.4 months, LOCRC 44.4 months). Cross-cohort survival comparisons are not appropriate due to different OS origin definitions.

### 3.5. Cox regression analysis

Multivariate Cox regression results are presented in Table 3 and Fig 4. In the TCGA discovery cohort adjusted for tumor stage (n = 503, events = 98), only tumor stage III-IV was independently prognostic (HR = 2.52; 95% CI 1.60 to 3.97; p < 0.001). No individual gene mutation achieved statistical significance in the stage-adjusted TCGA model, likely reflecting limited statistical power. In the MSK-IMPACT validation cohort adjusted for sample type (n = 4,091, events = 1,387), KRAS (HR = 1.23; 95% CI 1.10 to 1.38; p < 0.001), NRAS (HR = 1.38; 95% CI 1.08 to 1.75; p = 0.009), and BRAF (HR = 1.29; 95% CI 1.08 to 1.55; p = 0.005) mutations were independently associated with worse OS. MSI-H was independently associated with better OS (HR = 0.55; 95% CI 0.36 to 0.86; p = 0.008). Sample type (Metastatic vs. Primary) was the strongest independent predictor (HR = 1.70; 95% CI 1.53 to 1.89; p < 0.001), confirming that this variable should not be omitted from survival models. Age group (EOCRC vs LOCRC) was not an independent prognostic factor in either cohort.

**Table 3:** Multivariate Cox regression for overall survival with confounder adjustment.

Variable	TCGA HR (95%CI)	p	MSK Full HR	p	MSK EOCRC-only HR	p
Age < 50 vs ≥ 50	1.86 (0.92-3.75)	0.083†	0.98 (0.88-1.10)	0.789	—	—
KRAS mutation	1.01 (0.67-1.54)	0.947	1.23 (1.10-1.38)	<0.001*	1.12 (0.92-1.36)	0.275
NRAS mutation	—	—	1.38 (1.08-1.75)	0.009*	1.11 (0.70-1.78)	0.652
BRAF mutation	0.85 (0.28-2.57)	0.777	1.29 (1.08-1.55)	0.005*	1.37 (0.99-1.88)	0.054†
TP53 mutation	1.33 (0.88-2.01)	0.174	0.97 (0.85-1.11)	0.665	0.91 (0.71-1.16)	0.435
MSI-H	1.33 (0.35-5.05)	0.678	0.55 (0.36-0.86)	0.008*	0.44 (0.11-1.78)	0.251
Stage III-IV (TCGA)	2.52 (1.60-3.97)	<0.001*	—	—	—	—
Metastatic sample (MSK)	—	—	1.70 (1.53-1.89)	<0.001*	1.77 (1.47-2.14)	<0.001*

LOCRC (≥50 years) is the reference category for age group. TCGA MV: n = 503, events = 98 (adjusted for Stage; complete-case analysis: 10 patients with missing stage and 23 patients with missing OS data were excluded). MSK Full MV: n = 4,091, events = 1,387 (adjusted for Sample Type). MSK EOCRC-only MV: n = 1,411, events = 452; age group not applicable as all patients are < 50y. *p < 0.05; †p < 0.10. HR: hazard ratio; CI: confidence interval; —: not included. In the EOCRC-only model, no gene mutation retains independent statistical significance, indicating that KRAS/NRAS/BRAF/MSI-H prognostic effects are general CRC biomarkers rather than EOCRC-specific.

**Fig 4:**
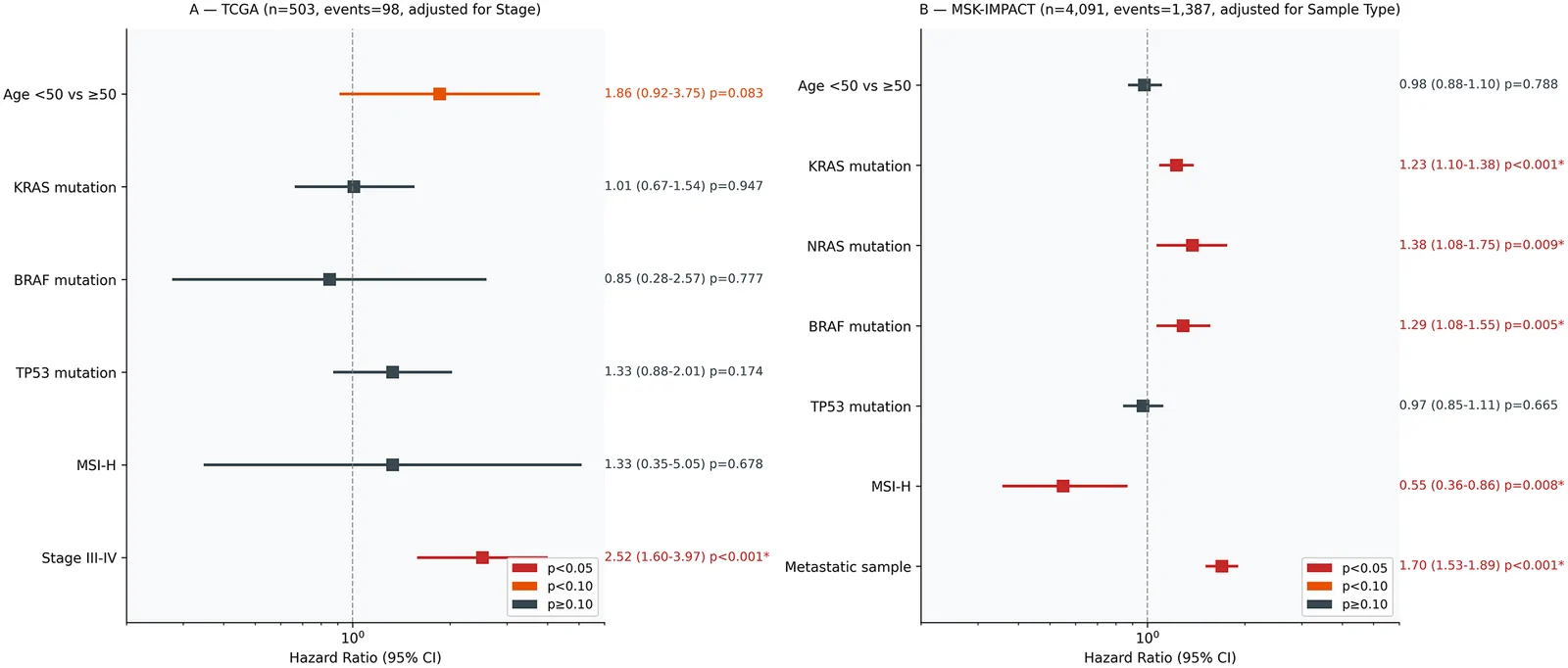
Forest plots of multivariate Cox regression. Left: TCGA discovery cohort (adjusted for Stage III-IV). Right: MSK-IMPACT validation cohort (adjusted for metastatic sample type). Red: p < 0.05; Orange: p < 0.10; Grey: p ≥ 0.10.

To directly address whether these prognostic effects are specific to EOCRC, we constructed an EOCRC-restricted multivariate model in the MSK-IMPACT cohort (n = 1,411, events = 452). In this analysis, KRAS (HR = 1.12; 95% CI 0.92 to 1.36; p = 0.275), NRAS (HR = 1.11; 95% CI 0.70 to 1.78; p = 0.652), BRAF (HR = 1.37; 95% CI 0.99 to 1.88; p = 0.054), TP53 (HR = 0.91; 95% CI 0.71 to 1.16; p = 0.435), and MSI-H (HR = 0.44; 95% CI 0.11 to 1.78; p = 0.251) all lost independent statistical significance, while metastatic sample type remained strongly prognostic (HR = 1.77; 95% CI 1.47 to 2.14; p < 0.001). This finding indicates that the prognostic effects of KRAS/NRAS/BRAF/MSI-H observed in the overall cohort are driven predominantly by the larger LOCRC subgroup and are not, in the strict statistical sense, EOCRC-specific.

## 4. Discussion

In this dual-cohort genomic analysis of nearly 4,800 CRC patients after exclusion of MMR-mutated tumors, we make three principal contributions to the EOCRC literature. First, we confirm BRAF mutation depletion in EOCRC across two independent cohorts, but demonstrate that this difference is substantially attenuated after stratification by tumor location, suggesting that at least part of the BRAF-EOCRC association reflects differential tumor location distribution rather than a purely age-onset-specific biological effect. Second, we validate KRAS, NRAS, BRAF, and MSI-H as CRC prognostic biomarkers in a real-world clinical NGS cohort adjusted for a stage-related proxy (sample type). Third, and importantly, we demonstrate through an EOCRC-restricted multivariate analysis that these biomarker prognostic effects are not specific to EOCRC, but rather appear to be general CRC prognostic biomarkers driven predominantly by the larger LOCRC subgroup.

The observation that BRAF (any) and BRAF V600E specifically are less common in EOCRC is well-established in the literature [7–10] and reflects the known biology of BRAF V600E-mutant CRC as predominantly arising via the serrated pathway in the proximal colon of older patients. Our contribution is showing that after location stratification, the BRAF-EOCRC differences within each tumor location subgroup are considerably smaller and non-significant. This does not refute the association but reframes it: EOCRC-BRAF depletion appears to be, at least in substantial part, a downstream consequence of EOCRC patients having proportionally more distal and rectal tumors, where BRAF mutation is already uncommon regardless of age.

The lack of EOCRC-specific independent prognostic effect for KRAS, NRAS, and BRAF is clinically important. These biomarkers are important CRC prognostic and predictive markers, and they should continue to be tested in all CRC patients, including EOCRC, for their well-established therapeutic implications (anti-EGFR eligibility and treatment selection in RAS-mutated disease [13], BRAF-targeted therapy, and immune checkpoint inhibitor eligibility for MSI-H tumors [14]). However, our findings suggest that within an EOCRC-only population, these mutations should not be interpreted as carrying additional prognostic weight beyond their role as general CRC biomarkers. This nuance is consistent with the broader oncologic principle that biomarker effects observed in general CRC cohorts should not be assumed to generalize identically to younger patients.

The discordant survival findings between our two cohorts require careful interpretation, particularly in light of the OS-measurement origin difference and referral bias. The MSK-IMPACT cohort measures OS from sample collection date. Sequencing at MSK-IMPACT is typically performed at the time of recurrence, progression, or metastatic biopsy to identify actionable targets, which introduces both lead-time bias (patients who survive long enough to be sequenced are, by definition, still alive at that time) and immortal-time bias. Younger EOCRC patients may be sequenced earlier in their disease trajectory (for example, at initial diagnosis with molecular characterization), while older LOCRC patients may be sequenced at later disease points. Consequently, the apparently favorable OS of EOCRC in MSK-IMPACT (49.4 vs 44.4 months) should be interpreted purely as “survival following clinical molecular sequencing,” not as natural-history survival. This interpretation is supported by our observation in TCGA, where EOCRC patients had significantly more advanced stage disease (Stage III-IV 62.7% vs 42.6%, p = 0.003), which would be expected to yield worse, not better, OS in a true natural-history comparison. Prior population-based analyses have similarly reported worse or comparable OS in EOCRC compared to average-onset CRC in general population cohorts [9,15].

The strong independent prognostic effect of metastatic sample type (HR = 1.70) in the MSK-IMPACT multivariate model validates the use of this variable as a stage-related proxy. Prior EOCRC genomic analyses that did not adjust for sample type or stage in the MSK-IMPACT cohort would be expected to have inflated apparent biomarker effect sizes and biased survival comparisons.

Several limitations warrant acknowledgment. First, tumor stage data are unavailable in MSK-IMPACT; sample type is a useful but imperfect proxy. Second, treatment data are unavailable in both cohorts, which limits interpretation of the survival findings: MSI-H patients may have received immunotherapy, and the survival advantage observed for MSI-H may reflect a combination of biological and treatment effects. Third, the OS definition differs between cohorts (diagnosis vs sample collection), precluding direct cross-cohort survival comparisons. Fourth, tumor location categorization from ICD-O-3 or PRIMARY_SITE is imperfect and may not fully capture the biological differences between right- and left-sided CRC. Fifth, our exclusion strategy targeted MMR-mutated tumors identified through somatic sequencing. This captures Lynch cases through the two-hit mechanism (in which the tumor acquires a somatic mutation in the same MMR gene as the germline defect) but may miss Lynch patients whose tumors did not acquire a second-hit somatic MMR mutation in the analyzed sample. Additionally, MSI-H tumors caused by sporadic MLH1 promoter hypermethylation (without MMR gene mutation) remain in the analysis cohort, as methylation data are not available in cBioPortal for these studies. Sixth, all mutation-frequency p-values are nominal and were not corrected for the 13 simultaneous comparisons performed per cohort. Seventh, the TCGA EOCRC subgroup remains small (n = 67 after MMR-mut exclusion), limiting statistical power. Eighth, sample-collection-based OS in MSK-IMPACT introduces potential lead-time and immortal-time bias, extensively discussed above. Ninth, MSK-IMPACT is a tertiary-center cohort and may not represent population-level CRC epidemiology.

## 5. Conclusions

In this dual-cohort analysis of 4,790 CRC patients after exclusion of MMR-mutated tumors, BRAF mutation depletion in EOCRC is confirmed but is substantially attenuated within tumor location strata, indicating that the effect is at least partially explained by tumor location distribution. KRAS, NRAS, BRAF, and MSI-H are validated as CRC prognostic biomarkers in a real-world clinical NGS cohort adjusted for sample type as a stage-related proxy. Critically, these biomarker prognostic effects are not independently significant within the EOCRC subgroup alone, indicating that they represent general CRC prognostic markers rather than EOCRC-specific biomarkers. These findings support continued comprehensive NGS profiling in EOCRC for therapeutic guidance while cautioning against interpreting general CRC prognostic biomarkers as carrying additional EOCRC-specific prognostic weight. The methodological framework developed here (MMR-mutated tumor exclusion, tumor location stratification, sample-type adjustment, and EOCRC-restricted analysis) provides a template for future EOCRC genomic epidemiology.
